# Appendiceal Orifice Inflammation in Ulcerative Colitis Mimicking Mucosa-Associated Lymphoid Tissue Lymphoma in the Cecum

**DOI:** 10.1155/2020/8893604

**Published:** 2020-10-07

**Authors:** Masaya Iwamuro, Takahide Takahashi, Takehiro Tanaka, Tomohiro Toji, Sakiko Hiraoka, Seiji Kawano, Yoshiro Kawahara, Hiroyuki Okada

**Affiliations:** ^1^Department of Gastroenterology and Hepatology, Okayama University Graduate School of Medicine, Dentistry, and Pharmaceutical Sciences, Okayama 700-8558, Japan; ^2^Division of Medical Support, Okayama University Hospital, Okayama 700-8558, Japan; ^3^Department of Pathology, Okayama University Graduate School of Medicine, Dentistry, and Pharmaceutical Sciences, Okayama 700-8558, Japan; ^4^Department of Practical Gastrointestinal Endoscopy, Okayama University Hospital, Okayama 700-8558, Japan

## Abstract

A 55-year-old Japanese woman, who had been diagnosed with ulcerative colitis at 18 years of age, underwent screening endoscopy examinations. Esophagogastroduodenoscopy revealed an extranodal marginal zone lymphoma of mucosa-associated lymphoid tissue (MALT lymphoma) of the stomach. Colonoscopy showed a slightly elevated reddish lesion with dilated microvessels but no erosions or ulcers. Although MALT lymphoma in the cecum was endoscopically suspected, flow cytometry and pathological analyses led to the diagnosis of appendiceal orifice inflammation in ulcerative colitis. This case highlights the diversity of the endoscopic appearance of appendiceal orifice inflammation in ulcerative colitis.

## 1. Introduction

Ulcerative colitis typically causes continuous inflammation in the colorectal mucosa extending from the rectum to the more proximal colon. However, the peri-appendiceal mucosa in the cecum is sometimes involved in a skip lesion [1–5]. This type of cecal lesion is referred to as “appendiceal orifice inflammation,” “peri-appendiceal patch,” or “cecal patch.” The colonoscopic features of appendiceal orifice inflammation include mucosal erythema, granularity, erosion/ulceration, and friability [1].

We, herein, report a case involving a patient who had ulcerative colitis concomitant with extranodal marginal zone lymphoma of mucosa-associated lymphoid tissue (MALT lymphoma) in the stomach. Of note, although the patient's cecal lesion was initially considered to be involved with the MALT lymphoma, flow cytometry and pathological analyses led to the diagnosis of appendiceal orifice inflammation in ulcerative colitis. Typical endoscopic features of colorectal MALT lymphoma and appendiceal orifice inflammation in ulcerative colitis are discussed later in this report.

## 2. Case Presentation

**Figure 1:**
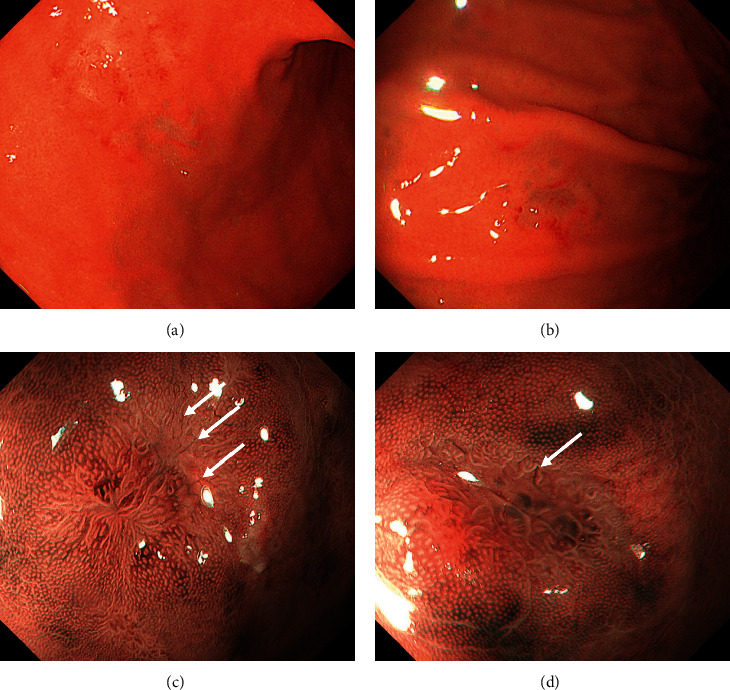
Esophagogastroduodenoscopy images. Multiple white irregularly shaped depressed lesions with partial redness are seen in the gastric body (a, b). Magnified narrow-band imaging images show the disappearance of the pit structure and elongated microvessels within the depressed areas (c, d, arrows). These microstructures are typical of gastric MALT lymphoma. MALT: mucosa-associated lymphoid tissue.

**Figure 2:**
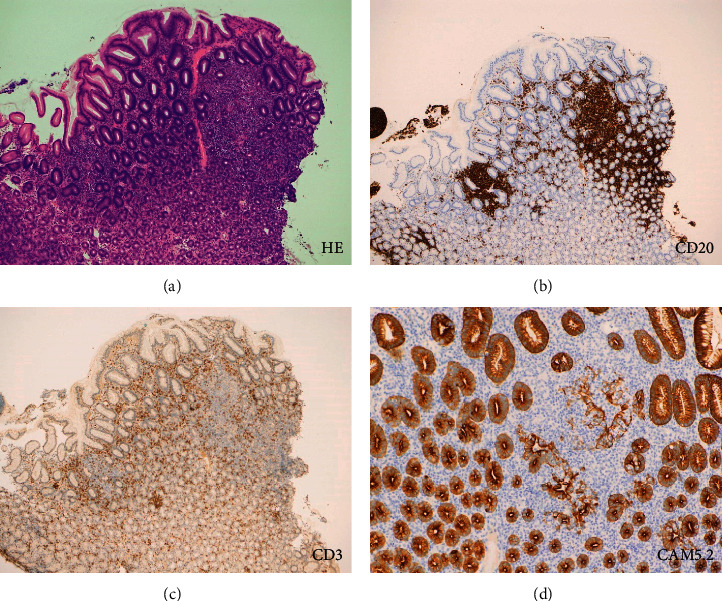
Images of the pathologic analysis of the gastric lesion. Small to medium sized lymphoid cells are observed in the biopsy specimens (a). These cells are positive for CD20 (b) and negative for CD3 (c). Immunostaining for cytokeratin (CAM5.2) shows the formation of lymphoepithelial lesions (d). Gastric MALT lymphoma was diagnosed.

A 55-year-old Japanese woman underwent screening esophagogastroduodenoscopy. The patient had been diagnosed with ulcerative colitis at the age of 18 years. Since palmoplantar pustulosis and arthritis of the sternoclavicular and foot joints appeared after anti-tumor necrosis factor-*α* antibody use, her ulcerative colitis was treated with a mesalazine and *Clostridium butyricum* preparation. She had also been taking fexofenadine, tranexamic acid, and brotizolam for sleep disturbance and prevention of drug allergy. She had a history of *Helicobacter pylori* eradication. Esophagogastroduodenoscopy revealed multiple white irregularly-shaped depressed lesions with partial redness in the gastric body (Figures 1(a) and 1(b)). Narrow-band imaging with magnification showed disappearance of the pit structure and elongated microvessels within the depressed areas (Figures 1(c) and 1(d), arrows), which are the typical features of gastric MALT lymphoma. Biopsy of the gastric lesion revealed the infiltration of small- to medium-sized lymphoid cells (Figure 2(a)), which were positive for CD20 (Figure 2(b)) and negative for CD3 (Figure 2(c)). Immunostaining for cytokeratin (CAM5.2) showed the formation of lymphoepithelial lesions (Figure 2(d)). BIRC3-MALT1 translocation was detected in the neoplastic cells. Lymphoma cells were not detected in the bone marrow on bone marrow aspiration and biopsy examinations. Computed tomography scanning with contrast media showed no lymphadenopathy. Consequently, gastric MALT lymphoma with *t*(11;18)(q21;q21) was diagnosed.

**Figure 3:**
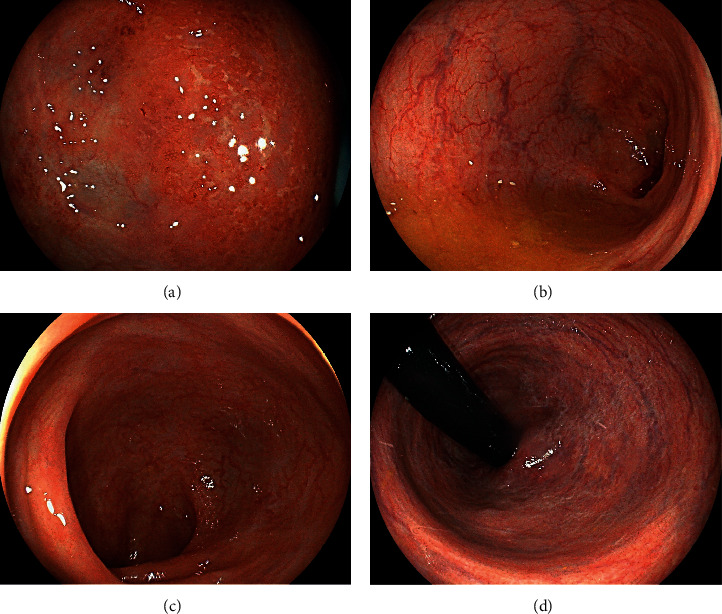
Colonoscopy images. Coarse reddish mucosa with yellowish-white pus-like deposits is observed in the splenic flexure (a). Atrophic mucosa with multiple ulcer scars is also seen in the descending to sigmoid colon region (b–d). These endoscopic findings are consistent with those for ulcerative colitis.

**Figure 4:**
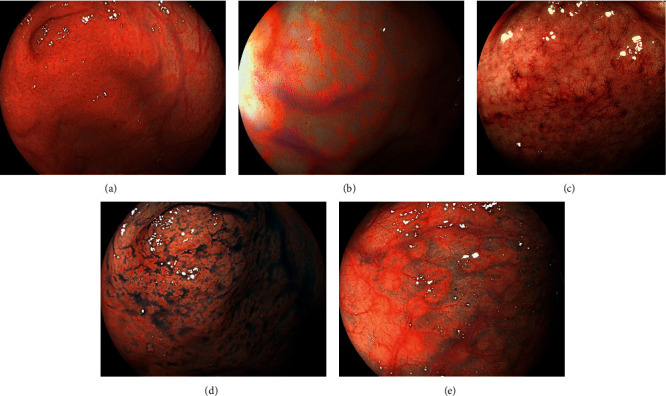
Colonoscopy images of the cecum. A reddish flat elevated lesion is seen (a). Magnified linked color imaging image shows elongated microvessels (b). Partial disappearance of the pit structure and elongated microvessels are observed in the narrow-band imaging image (c). Indigo carmine spraying reveals that the areas with no pit structures are slightly depressed (d). Papular lesions resembling lymphoid follicles are observed in the cecum and ascending colon (e). Although we initially considered this lesion to be involved with the MALT lymphoma, the final diagnosis was appendiceal orifice inflammation in ulcerative colitis.

**Figure 5:**
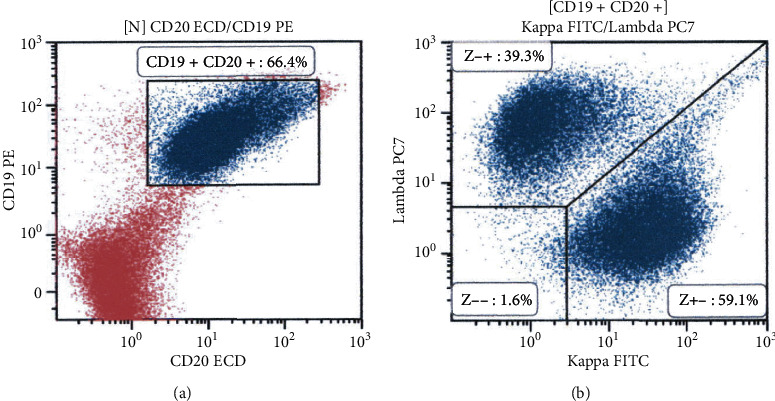
Flow cytometry analysis results. We used an endoscopic biopsy specimen for flow cytometry analysis; light chain restriction was not observed. CD19^+^ and CD20^+^ cells are gated (a), and light chain expression analysis reveals an almost equal number of Ig *κ*- and Ig *λ*-producing cells (b).

**Figure 6:**
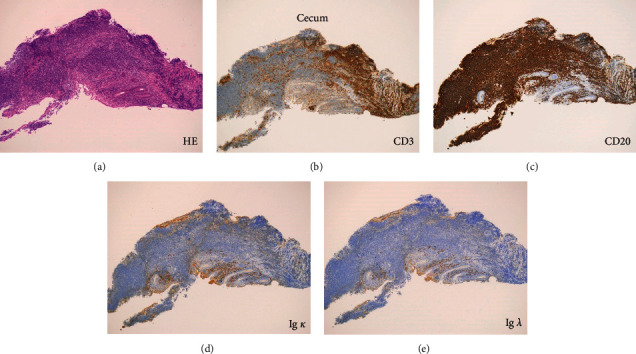
Images of the pathologic analyses of the cecal lesion. Inflammatory cell infiltration, mainly consisting of mononuclear cells, in addition to some neutrophils is observed in the biopsy specimens (a). Although CD20-positive lymphocytes are predominant (b), CD3-positive cells are observed as well (c). Predominance of neither Ig *κ* (d) nor Ig *λ* (e) is observed on staining.

Colonoscopy revealed coarse reddish mucosa with yellowish-white pus-like deposits in the splenic flexure (Figure 3(a)), which are the representative endoscopic features of ulcerative colitis. Atrophic mucosa with multiple ulcer scars was observed in the descending to the sigmoid colon region, consistent with the characteristics of ulcerative colitis in the remission phase. In addition, a reddish flat elevated lesion was observed in the cecum (Figure 4(a)). Linked color imaging with magnification showed elongated microvessels (Figure 4(b)). The partial disappearance of the pit structure and elongated microvessels were observed on narrow-band imaging (Figure 4(c)). Indigo carmine spraying revealed that the areas with no pit structures were slightly depressed, as shown by the pooling of the indigo carmine dye (Figure 4(d)). Papular lesions resembling the lymphoid follicles were observed in the cecum and ascending colon (Figure 4(e)). Due to the similarity of the magnifying endoscopic features between the cecal lesion and the gastric MALT lymphoma, we initially considered the cecal lesion to be involved with the MALT lymphoma. However, flow cytometry analysis using an endoscopic biopsy specimen showed no light chain restriction (Figure 5). On pathological analysis of the biopsied specimens, inflammatory cell infiltration, mainly consisting of mononuclear cells, in addition to some neutrophils was observed (Figure 6). Although the CD20-positive lymphocytes were predominant, the CD3-positive cells were observed as well. Ig *κ* and Ig *λ* predominance was not observed on staining, which was consistent with the results of the flow cytometry assay. Thus, we diagnosed the cecal lesion as appendiceal orifice inflammation in ulcerative colitis, rather than being involved with the lymphoma. After discussing with the patient, we decided to manage her gastric MALT lymphoma with a watch-and-wait approach because the lesion was localized in the stomach, and she showed no symptoms, bulky adenopathy, or organ compromise.

## 3. Discussion

Skip inflammation of the peri-appendiceal region in patients with ulcerative colitis was first described in 1958 by Lumb and Protheroe [1, 6]. Appendiceal orifice inflammation is usually observed during colonoscopy as mucosal erythema, granularity, erosion/ulceration, and friability, all features essentially similar to those observed for continuous inflammation extending proximally from the rectum in ulcerative colitis [1]. In the present patient, a segmental skip lesion showing coarse reddish mucosa with yellowish-white pus-like deposits was observed in the splenic flexure (Figure 3(a)), while reddish flat elevated lesions were observed in the peri-appendiceal region; the skip lesion showed the typical endoscopic features of ulcerative colitis, but the latter did not.

MALT lymphoma generally develops in the stomach, and colorectal involvement in MALT lymphoma is rare. Jeon et al. investigated the endoscopic features of 51 patients with colorectal MALT lymphoma and classified the lesions as subepithelial tumors (51%), polyps (20%), epithelial masses (14%), and ileitis (16%). MALT lymphoma was most frequently observed in the rectum (39%), followed by the ileocecal area (30%) [7]. Won et al. reviewed the data for 73 patients with colorectal MALT lymphomas, 67 of whom were previously reported cases in the literature [8]. Although the endoscopic features were not categorized in detail, the MALT lymphomas were primarily observed as a single polypoid lesion (70%), while multiple polypoid lesions were also observed (30%). These tumors were located in the rectum (74%), right colon (14%), transverse colon (4%), and sigmoid colon (8%). Therefore, a single protruded lesion of a subepithelial tumor or polypoid appearance is the most commonly observed morphology of colorectal MALT lymphomas, which generally involve the rectum, followed by the right colon. However, the endoscopic features of colorectal MALT lymphomas can vary from patient to patient [9–15].

Seo et al. reported a patient with a MALT lymphoma in the transverse colon, which showed a flat elevated lesion with diffuse granularity of the mucosa and loss of vascularity. Narrow-band imaging revealed an abnormal branch-like capillary pattern in patches [16]. These macroscopic appearances were similar to those of the present patient's cecal lesion. In addition, the features of the cecal lesion of the present patient resembled those of her gastric MALT lymphoma lesions, as observed on narrow-band imaging with magnification; both lesions showed disappearance of the pit structure and elongated microvessels within the depressed areas (Figures 1(c), 1(d), and 4(c)). Based on the similarity in characteristics with those of the gastric MALT lymphoma lesion and of the previously reported patient with colonic MALT lymphoma and the dissimilarity with the typical features of ulcerative colitis, we initially considered the cecal lesion to be involved with the MALT lymphoma. However, the pathological diagnosis was appendiceal orifice inflammation, rather than a neoplastic lesion. These results underscore that appendiceal orifice inflammation in ulcerative colitis may present various morphologies and masquerade as a lymphoma.

For the present patient, flow cytometry analysis performed using an endoscopic biopsy specimen aided in establishing the prompt nonlymphoma diagnosis. Flow cytometric immunophenotyping is a sensitive and accurate method for the diagnosis of lymphomas that enables the detection and characterization of neoplastic lymphocytes. Since B-cell lymphomas generally develop due to the unrestricted growth of a particular clone of B cells, neoplastic cells express only one class of immunoglobulin light chain, which is either a *κ* or *λ* chain. Thus, light chain restriction is the hallmark of B-cell lymphomas. For the present patient, the flow cytometry results were obtained on the day of colonoscopy, resulting in the timely achievement of the nonlymphoma diagnosis.

In Japan, flow cytometry has not typically been used to diagnose gastrointestinal lymphoma, as an appropriate protocol has not yet been established for lymphocyte isolation from endoscopic biopsy specimens. We recently established lymphocyte isolation techniques and used them to confirm that light chain expression analysis can be performed on such specimens [17, 18]. However, even at our facility, only a limited number of endoscopists are able to perform this analysis using our newly developed methods. Therefore, flow cytometry was not performed with gastric MALT lymphoma lesion samples. Nevertheless, we anticipate that our lymphocyte isolation technique will be widely used in the future for routine clinical diagnosis of gastrointestinal lymphoma.

In conclusion, this case highlights the diversity of the endoscopic appearance of appendiceal orifice inflammation in ulcerative colitis. Although pathological analysis is essential for the differentiation of nonlymphoma lesions from lymphomas, flow cytometry analysis has the advantage of delivering results rapidly.

## Data Availability

Access to data is restricted.
